# Lnc2Catlas: an atlas of long noncoding RNAs associated with risk of cancers

**DOI:** 10.1038/s41598-018-20232-4

**Published:** 2018-01-30

**Authors:** Chao Ren, Gaole An, Chenghui Zhao, Zhangyi Ouyang, Xiaochen Bo, Wenjie Shu

**Affiliations:** Department of Biotechnology, Beijing Institute of Radiation Medicine, Beijing, China

## Abstract

Lnc2Catlas (http://lnc2catlas.bioinfotech.org/) is an atlas of long noncoding RNAs (lncRNAs) associated with cancer risk. LncRNAs are a class of functional noncoding RNAs with lengths over 200 nt and play a vital role in diverse biological processes. Increasing evidence shows that lncRNA dysfunction is associated with many human cancers/diseases. It is therefore important to understand the underlying relationship between lncRNAs and cancers. To this end, we developed Lnc2Catlas to compile quantitative associations between lncRNAs and cancers using three computational methods, assessing secondary structure disruption, lncRNA-protein interactions, and co-expression networks. Lnc2Catlas was constructed based on 27,670 well-annotated lncRNAs, 31,749,216 SNPs, 1,473 cancer-associated proteins, and 10,539 expression profiles of 33 cancers from The Cancer Genome Atlas (TCGA). Lnc2Catlas contains 247,124 lncRNA-SNP pairs, over two millions lncRNA-protein interactions, and 6,902 co-expression clusters. We deposited Lnc2Catlas on Alibaba Cloud and developed interactive, mobile device-compatible, user-friendly interfaces to help users search and browse Lnc2Catlas with ultra-low latency. Lnc2Catlas can aid in the investigation of associations between lncRNAs and cancers and can provide candidate lncRNAs for further experimental validation. Lnc2Catlas will facilitate an understanding of the associations between lncRNAs and cancer and will help reveal the critical role of lncRNAs in cancer.

## Introduction

Long noncoding RNAs (lncRNAs), a class of noncoding RNAs with lengths exceeding 200 nucleotides, play a vital role in dysfunctional networks in cancer^1^. Increasing evidence suggests that single nucleotide polymorphisms (SNPs) in lncRNAs may alter their functions and induce cancer^2–4^. In addition, lncRNAs can silence other gene loci by interacting with diverse genes or acting as sponges in binding processes^5–7^. Moreover, overexpressed lncRNAs in tumour cells correlate with genes that impact cell cycle regulation, survival, pluripotency, immune responses or other functions^8^. Understanding the underlying relationship between lncRNAs and cancer is an important task^9^.

Many databases compiling lncRNAs have been developed and provide abundant resources for exploring the function of lncRNAs. LncRNAdb provides comprehensive annotations of eukaryotic long non-coding RNAs^10^. LNCipedia is an integrated database that contains annotated human lncRNA transcripts obtained from different sources^11^. NONCODE provides non-coding RNAs across 17 species^12^. LncATLAS is a comprehensive resource for information on lncRNA localization in cells based on human RNA-sequencing data^13^. In a recent study, *Hon CC et al*. used FANTOM5 cap analysis of gene expression data to generate a reliable long non-coding RNA (lncRNA) dataset across 1,829 human samples^14^. Several resources that indicate associations between lncRNAs and diseases/cancers have been constructed and have contributed to studies on the interactions of lncRNAs and cancer. LincSNP links disease-associated SNPs to human lincRNAs and identifies disease-associated SNPs in lncRNA transcription factor binding sites (TFBSs)^15^. LncRNASNP identifies SNPs in lncRNAs and analyses their potential impacts on lncRNA structure and function^16^. LncRNADisease curates and annotates 478 entries of experimentally supported lncRNA-disease associations, including 128 human lncRNAs and 166 diseases^17^. The LncRNADisease database also curates lncRNA interaction partners at various molecular levels. Lnc2Cancer provides more than 1,000 manually curated associations between 531 lncRNAs and 86 human cancers^18^. TANRIC represents a resource for exploring the function and clinical relevance of lncRNAs in 20 cancer types^19^. lnCaNet analyses the interactions between 9,641 lncRNAs and 2,544 cancer genes and provides 8,494,907 significant co-expression pairs^20^. However, a database compiling quantitative associations between lncRNAs and cancer from distinct aspects is still needed.

In this study, we developed Lnc2Catlas to compile the underlying quantitative associations between lncRNAs and cancer using multiple methods and data sources. Lnc2Catlas assesses lncRNA-cancer associations with three quantified ranking methods, assessing secondary structure disruptions, lncRNA-protein interactions, and co-expression networks. Lnc2Catlas was constructed based on 27,670 well-annotated lncRNAs, 31,749,216 SNPs, 1,473 cancer-associated proteins, and 10,539 expression profiles of 33 cancers from The Cancer Genome Atlas (TCGA, https://portal.gdc.cancer.gov/). It contains 247,124 lncRNA-SNP pairs, over two millions lncRNA-protein interactions, and 6,902 co-expression clusters. We deposited Lnc2Catlas on Alibaba Cloud and developed interactive, mobile device-compatible, user-friendly interfaces to assist users in searching and browsing with ultra-low latency. Lnc2Catlas is a valuable resource for investigating the associations between lncRNAs and cancer from distinct aspects. Lnc2Catlas is now freely available at http://lnc2catlas.bioinfotech.org.

## Results

### Database interface

**Figure 1:**
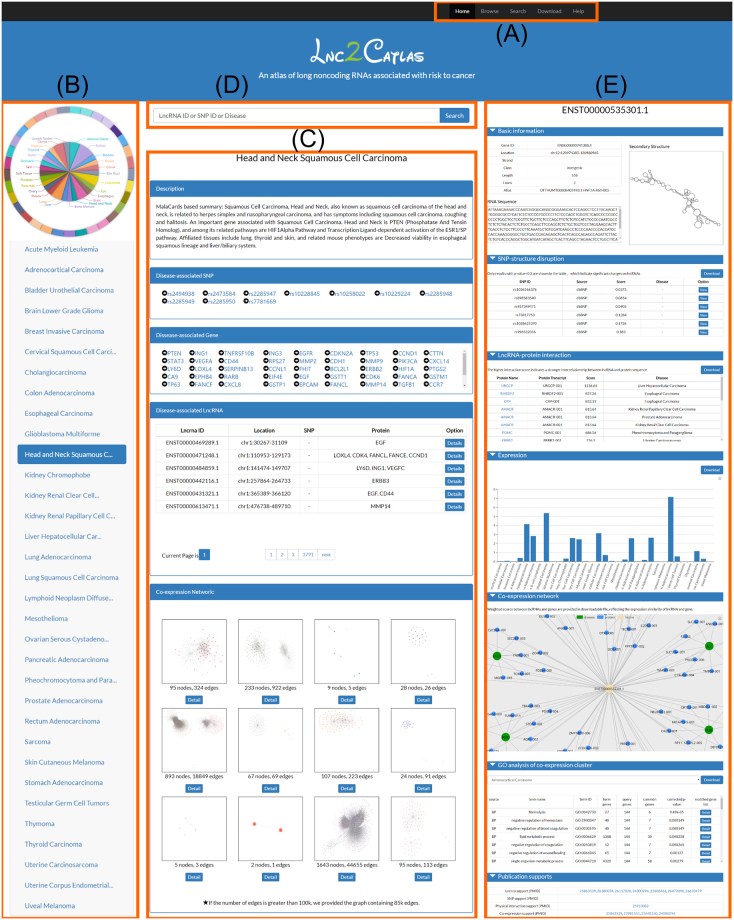
Screenshots of the Lnc2Catlas web interface. (**A**) Navigation bar at the top of the pages; (**B**) pie chart on the ‘Home’ page and the list of cancers collected in Lnc2Catlas on the ‘Browse’ page; (**C**) browse page for a certain cancer; (**D**) search box provided on the ‘Home’ and ‘Search’ pages; and (**E**) detail page of a queried lncRNA.

Lnc2Catlas provides browse and search functions for users to query the database, and these functions are accessed from the top navigation bar (Fig. 1A). All data in the database can be obtained on the ‘Download’ page. User-defined content on the detail pages for each lncRNA is also provided for download. Detailed help materials are available on the ‘Help’ page to ensure easy use for first-time visitors.

We have provided direct entry to all 33 cancers collected by Lnc2Catlas. Users can select a cancer type on the left of the ‘Browse’ page or can tap on a region of the pie chart on the ‘Home’ page (Fig. 1B). For each cancer, the ‘Browse’ page displays a brief description of the cancer and cancer-associated SNPs, proteins, and lncRNAs obtained using the three methods indicated above. In addition, each SNP and protein are linked to their corresponding pages in dbSNP^21^ and GeneCards^22^, respectively. Clicking the corresponding detail button in the lncRNA list will redirect the user to the lncRNA detail page. In addition, thumbnail blocks of the weighted co-expression network generated through weighted gene co-expression network analysis (WGCNA)^23^ are shown. Users can click the detail button to zoom in their view of the network and can select a node of interest to see its connected lncRNAs and proteins. Additionally, users can click the lncRNA ID in the dialog box to be redirected to the lncRNA detail page. Only the 85,000 most-weighted connections are presented in each network due to memory and time costs, but the full-scale network can still be accessed on the ‘Download’ page for further research (Fig. 1C).

The search box accepts case-insensitive inputs to query Lnc2Catlas using different identifiers on the ‘Search’ and ‘Home’ pages (Fig. 1D). Three types of input are supported: SNP (*rs* ID), protein (Gene Symbol) and lncRNA transcript ID (ENST ID). For each SNP input, Lnc2Catlas returns lncRNAs ranked by *p*-values based on secondary structure disruption analysis. The SNP data source and associated cancer are also presented in a tabular manner. For each protein input, the returned results are presented similarly to those of a SNP query; the protein’s associated lncRNAs ranked by Global Score and its related cancer are listed. For lncRNA inputs, the case is more complicated. If the query returns multiple results, two tables are displayed, listing lncRNA-SNP interactions and lncRNA-protein interactions. In contrast, if the query returns a definite lncRNA, the page will directly redirect the user to the corresponding lncRNA detail page.

On the lncRNA detail page, Lnc2Catlas displays basic information on the lncRNA and the computational results obtained through secondary structure disruption analysis, lncRNA-protein interaction analysis, expression analysis, co-expression network analysis, and gene ontology (GO) and pathway analysis of co-expression clusters. A local base pair probability matrix and secondary structures are shown when users select a SNP in the SNP-structure disruption table. The expression value of the corresponding cancer is shown when the mouse is moved over the bar, providing an interactive view of the results. Moreover, the graph of the co-expression network can be scaled and dragged, and any node of the network and its adjacent nodes can be highlighted by hovering over it (Fig. 1E). In the GO and pathway analysis results, users can select a cancer type in the drop-down box to obtain term types, enrichment *p*-values and matched gene lists. The publications experimentally supporting the above lncRNA-cancer associations are presented in the bottom of the detail page.

### Secondary structure disruption

Distinctive secondary structures are a primary prerequisite for the function of noncoding RNAs; therefore, alterations caused by disease-associated SNPs confer a predisposition to dysfunction and the possibility of severe disease. We used RNAsnp^24^ to quantitatively evaluate the impacts of the disruptions caused by SNPs to lncRNA secondary structure. In total, 19,745 lncRNA transcripts were found to be significantly altered by 247,124 dbSNPs. Among these lncRNA transcripts, 298 were defined as significantly altered by 914 cancer-associated SNPs. In this analysis, the secondary structures of all lncRNA transcripts and the local base pair probability matrix and secondary structures of the wild-type and mutant type of each lncRNA are presented in the detail page.

### LncRNA-protein interactions

The identification of interactions between lncRNAs and proteins through experimental methods is challenging. Thus, a database quantifying the strength of interaction between lncRNAs and cancer-associated proteins is extremely beneficial. To prioritize candidate lncRNA-protein pairs in cancer, we used Global Score^25^ to calculate binding scores between 27,670 lncRNA transcripts and 1,040 cancer-associated proteins. For each cancer type, only the expressed lncRNAs and proteins with fragments per kilobase million (FPKM) values > 0 were selected to calculate the binding interaction scores using Global Score. A higher score indicates a greater binding interaction strength between a lncRNA and a cancer-associated protein. Due to the large number of computations, Lnc2Catlas currently compiles over two million lncRNA-protein interactions, covering 27,670 lncRNA transcripts, with at least the top three genes for each cancer. All of these interactions are available in Lnc2Catlas.

### Co-expression network

LncRNAs are expressed in a tissue-specific and heterogeneously regulated manner in tumour/normal cells. The underlying regulatory expression patterns correlated with genes with key roles in cancer were explored by constructing weighted co-expression networks based on 10,539 expression profiles from TCGA through WGCNA. For each cancer, the co-expression network calculated via WGCNA was divided into small blocks with weighted edges between the lncRNAs and protein-coding genes. Twelve blocks on average were created for each cancer. In total, 395 blocks, ranging from 2,831 lncRNAs x 1,549 protein-coding genes in kidney chromophobe cancer to 3 lncRNAs x 2 protein-coding genes in breast invasive carcinoma, are presented in Lnc2Catlas. The results of co-expression network analysis were presented as clusters (modules) of highly correlated lncRNAs and genes for each cancer. In total, 6,902 clusters were defined in 33 cancer types, ranging from 59 clusters for breast invasive carcinoma to 348 clusters for brain lower-grade glioma. Further GO analysis of the 6,902 clusters in 33 cancer types revealed that 2,415 clusters were enriched with various GO and pathway terms.

### Case studies

**Figure 2:**
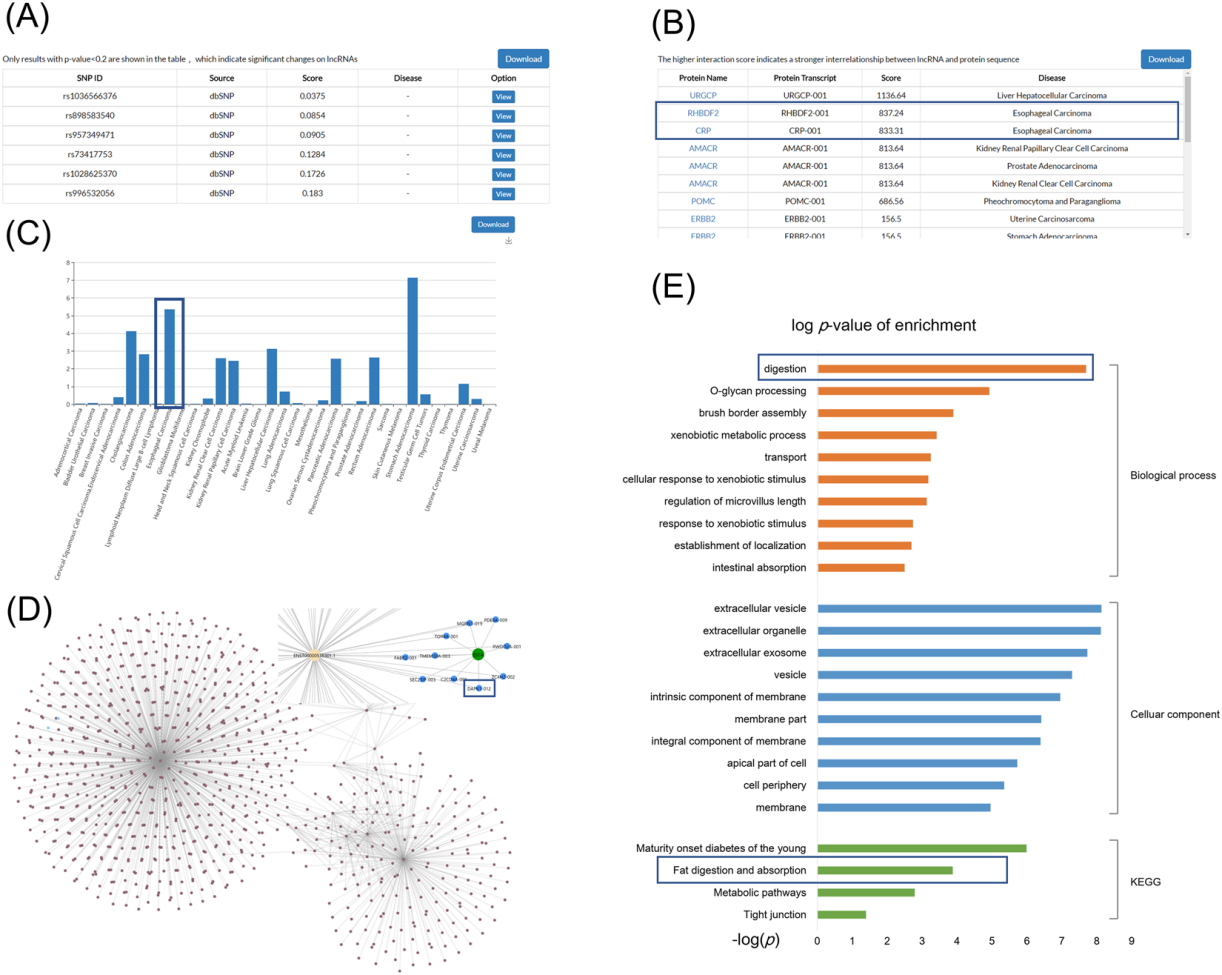
An example illustrating how Lnc2Catlas helps reveal the potential function of a lncRNA in oesophageal cancer. (**A**) A SNP located in the ENST00000535301.1 transcript significantly impacts secondary structure; (**B**) genes (RHBDF2 and CRP) with key roles in oesophageal cancer were highly associated with the transcript by Global Score; (**C**) ENST00000535301.1 was expressed at a relatively higher level in oesophageal cancer; and (**D**) ENST00000535301.1 s was found to correlate with the death-related gene DAPK1 (indicated with a red box) in oesophageal cancer based on co-expression networks. (**E**) GO and pathway analysis of genes that were highly correlated with ENST00000535301.1 involved in the cluster derived from co-expression analysis of oesophageal adenocarcinoma.

Lnc2Catlas compiles quantitative associations between lncRNAs and cancer. Lnc2Catlas can aid in the investigation of the potential role of lncRNAs in cancer by exploring the associations between lncRNAs and cancer. Two lncRNA transcripts are taken as examples to demonstrate how to explore lncRNA-cancer associations using Lnc2Catlas. ENST00000535301.1 is a lncRNA transcript located on chromosome 12 whose knockdown greatly impacts the proliferation of oesophageal adenocarcinoma cells^26^. This lncRNA transcript can be accessed in Lnc2Catlas by searching for its ID (Fig. 2). Secondary structure disruption analysis showed that 6 SNPs greatly altered the secondary structure of this transcript (Fig. 2A). LncRNA-protein interaction analysis demonstrated that this lncRNA transcript showed high interaction scores with RHBDF2 and CRP, of 837.24 and 833.31, respectively. RHBDF2, also known as IRHOM2, has been reported to play a critical role in activating or downregulating the EGFR signalling pathway connected to oesophageal adenocarcinoma^27,28^ (Fig. 2B). The postoperative levels of CRP were used to predict prognosis after treatment of oesophageal adenocarcinoma^29^. In addition, this transcript was differentially expressed in 33 cancer types and exhibited the second-highest ranked expression in oesophageal cancer samples (Fig. 2C). Furthermore, weighted co-expression analysis showed that this transcript displays a similar expression pattern to DAPK1, which mediates programmed cell death and is associated with diverse cancers^30,31^ (Fig. 2D). GO and pathway analysis of the highly correlated clusters involving this transcript and its associated genes in oesophageal adenocarcinoma revealed enrichment for genes involved in the functions of digestion and pathways of fat digestion and absorption (Fig. 2E). Together, the results provided by Lnc2Catlas suggest that the ENST00000535301.1 transcript may play an important role in oesophageal cancer.

ENST00000422494.1, a lncRNA transcript located on chromosome 20, has been reported to exhibit a function related to papillary thyroid carcinoma^32^. We searched for this transcript in Lnc2Catlas to examine its association with thyroid cancer (Figure S3). We found 11 SNPs located in ENST00000422494.1 that significantly disrupted its secondary structure (Figure S3A). In addition, this transcript presented the highest expression associated with thyroid cancer, exhibiting 6-times higher expression than the second-ranked transcript (Figure S3B). Co-expression network analysis showed that in thyroid cancer cells, ENST00000422494.1 was expressed in the same pattern as MPPED2, which has been widely reported to be overexpressed and to affect the malignancy of lesions in thyroid tissues^33,34^ (Figure S3C). Further GO and pathway enrichment analysis revealed that genes in cluster from thyroid cancer are involved in the thyroid hormone signalling pathway. These results suggest that ENST00000422494.1 may function in thyroid carcinoma (Figure S3D). These two examples demonstrate that Lnc2Catlas can truly aid in the exploration of associations between lncRNAs and cancer and provide clues to the potential roles of lncRNAs in cancer.

## Discussion

Evidence has suggested that lncRNAs play a crucial role in cancer; however, the screening of candidate lncRNAs and definition of their function in specific cancers is still challenging. To this end, we present Lnc2Catlas, which compiles multiple quantitative assessments of the associations between lncRNAs and cancer to help researchers prioritize candidate lncRNAs. A study based on TCGA project demonstrated that 60% of dysregulated lncRNAs were specific to only one tumour^35^. These tumour-specific dysregulated lncRNAs participate in diverse regulatory process as regulators or regulatees. To help researchers infer the underlying functional lncRNAs in specific tumours, we link lncRNAs with disease-associated SNPs and cancer-associated proteins and genes. Lnc2Catlas integrates multiple resources and constructs lncRNA-centric networks that associate cancer-specific lncRNAs with SNPs, proteins and genes related to each cancer. The two presented case studies demonstrate that our Lnc2Catlas is helpful to complement the experimental design and validation processes in studies of lncRNA functions in cancer by prioritizing candidate lncRNAs based on three quantitative assessments.

Other groups have also constructed lncRNA-cancer association databases, such as lncRNASNP, LncRNADisease, Lnc2Cancer, TANRIC, and lnCaNet. A comparison between Lnc2Catlas and these similar databases is presented in Table 1, which demonstrates some strong advantages of Lnc2Catlas in comparison with these other databases. One of the advanced characteristics distinguishing Lnc2Catlas is the ability to explore the quantitative associations between lncRNAs and cancer from distinct aspects. In studies of lncRNA functions, the prioritization of candidate lncRNAs in the experimental design and validation processes is challenging. Lnc2Catlas adopts three scoring methods to quantify the associations between lncRNAs and cancer. Thus, Lnc2Catlas can help researchers to rank candidate lncRNAs and explore their potential roles in cancer, on the basis of secondary structure disruption, lncRNA-protein interactions, and co-expression networks. To enhance the experimentally supported ability of our Lnc2Catlas, we curated 1,038 publications related to lncRNA-cancer associations in Lnc2Catlas.

**Table 1:** Comparison between Lnc2Catlas and other five cancer-associated databases.

Database		LncRNADisease	Lnc2Cancer	lncRNASNP	TANRIC	lnCaNet	Lnc2Catlas
Data	Number of lncRNAs	914	531	17,436	12,727	9,641	27,670
	Number of diseases	329	86	28	20	11	33
	SNPs	GWAS	Not available	GWAS and LD analysis	Not available	Not available	GWAS, Clinvar and LD analysis
	Genes	Not available	Not available	Not available	Collected from publications	Collected from publications	MalaCard, DisGeNet, Human protein Atlas
	Gene expression	Not available	Not available	Not available	TCGA	TCGA	TCGA
	Publication support	Available	Available	Not Available	Not available	Not Available	Available
Analysis	Analysis based on SNPs	Based on genomic neighbourhood	Not available	Based on miRNA-lncRNA target	Not available	Not available	Based on secondary structure
	Analysis based on physical interaction with genes	Not available	Not available	Not available	Not available	Not available for lncRNA-protein interactions	Available for 27,670 lncRNAs and 1,759 cancer-related genes
	Analysis based on gene expression	Not available	Not available	Not available	Differential expression and correlation with clinical data	Co-expression network analysis	Weighted gene co-expression network analysis
	Computational result coverage	1,564 lncRNAs	Not available	17,436 lncRNAs	12,727 lncRNAs	9,641 lncRNAs	27,670 lncRNAs
Interface	Mobile device compatibility	No	No	Yes	No	No	Yes
	Graphic visualization	No	No	Yes	No	No	Yes

We will continually update the database by integrating up-to-date resources and methods that quantify the associations between lncRNAs and cancer. In summary, Lnc2Catlas will facilitate our understanding of the associations between lncRNAs and cancer and will aid in the investigation of the potential role of lncRNAs in cancer.

## Materials and Methods

### LncRNAs, SNPs, and proteins

The relationship between lncRNAs and cancers was explored by selecting 10,539 expression files from 10,237 samples of 33 cancer types from TCGA; this dataset included profiles of 15,899 lncRNA genes and 20,241 coding genes. Annotations of the corresponding 27,670 lncRNA transcripts were collected from GENCODE V22 (hg38) for consistency with the data protocol for TCGA. A total of 31,749,216 SNPs located in lncRNA transcripts were collected from dbSNP (build 147). Among these SNPs, 92,604 were defined as cancer-associated SNPs, including 941 SNPs from genome-wide association studies (GWAS)^36^, 39,558 ClinVar SNPs^37^ marked as “Pathogenic” or “Likely Pathogenic”, and 52,105 SNPs in strong linkage disequilibrium (LD) with GWAS or ClinVar SNPs (*r*^2^ > 0.8). We also collected 1,473 curated cancer-associated proteins from MalaCards^38^, DisGeNET^39^, and the Human Protein Atlas in a recent study^40^, with an average 45 proteins for each cancer, ranging from 4 proteins in uterine corpus endometrial carcinoma to 321 proteins in breast invasive carcinoma.

### Quantifying associations between lncRNAs and cancer

Three different scoring methods were used to quantify associations between lncRNAs and cancer in Lnc2Catlas (Table 2). To quantify the impacts of the SNPs on lncRNA secondary structure, we used RNAsnp to calculate the disruption of secondary structure between the wild type and the mutant type and to predict the significance of disruptive impacts using default parameters. For each lncRNA-SNP pair, a *p*-value was calculated to indicate the significance of the disruptive impact of the SNP on the lncRNA. Disruptions with *p*-values < 0.2 (default threshold of RNAsnp) are considered significant, indicating that the corresponding lncRNA is more likely to undergo major structural changes. The secondary structures of all lncRNA transcripts were calculated using RNAfold from the ViennRNA packages^41^. The local base pair probability matrix and secondary structures of the wild-type and mutant type of each lncRNA were generated using ViennRNA packages and VARNA software^42^, respectively. For the analysis of lncRNA-protein interactions, we calculated the potential binding strength between 27,670 lncRNA transcripts and 1,473 cancer-associated proteins from MalaCards and other databases using Global Score with the default parameters. For the analysis of co-expression networks, the expression profiles of tumour and normal samples were merged into one matrix to produce the topological overlap matrix for each cancer type, which had an average size of approximately 55,000 × 55,000. The topological overlap matrix was then divided into small blocks with weighted edges between the lncRNAs and protein-coding genes, composed of the clusters (modules) of highly correlated lncRNAs and genes. Furthermore, we used g:Profiler^43^ to investigate GO and pathway enrichment for each cluster, employing a threshold *p*-value of 0.05. The GO of biological processes, cellular components, and molecular functions and the pathways of KEGG and Reactome were used in the enrichment analysis.

**Table 2:** Data source and methods adopted in Lnc2Catlas.

	Secondary structure disruption	lncRNA-protein binding interaction	Co-expression network analysis
Method	RNAsnp	GlobalScore	WGCNA
Input	SNP and sequence of lncRNA	Sequences of lncRNAs and cancer-related proteins	Expression profiles of lncRNAs and genes in different cancer
Data Sources	31,749,216 SNPs from dbSNP; 941 cancer-related SNPs from GWAS; and 39,558 cancer-related SNPs from Clinvar	1,040 cancer-related proteins from Malacard; 664 cancer-related proteins from DisGeNet; and 55 genes from Human Protein Atlas. In total, 1,473 genes.	10,539 expression files from 10,237 samples of 33 cancer types
Results	*p*-value indicating the significance of the impact of secondary structure disruption	Score indicating the binding strength	Clusters (modules) of highly correlated lncRNAs and genes
Results statistic	19,745 lncRNAs significantly altered by 247,124 SNPs. 298 lncRNAs altered by 914 cancer-associated SNPs.	Over two million lncRNA-protein interactions	6,902 clusters in 33 co-expression networks of 33 cancer types. 2,415 clusters with enriched GO and pathway terms
Publication supports	113	144	905

### Experimentally validated support

To enhance the experimentally supported ability of Lnc2Catlas, publications addressing lncRNAs and cancer were curated from PubMed. First, we used “(lncRNA or long noncoding RNA) AND (cancer OR tumour OR neoplasms)” as keywords to search the PubMed database. Second, we manually curated these publications to identify lncRNA-cancer associations. We classified these publications into different classes to support the associations in Lnc2Catlas. Publications describing the study of variants were used to support the existence of SNPs in lncRNAs in tumour/cancer samples. Publications addressing lncRNA-protein binding interactions were used to support the prediction of lncRNA-protein interactions. Publications reporting investigations of the expression pattern of lncRNAs and genes in tumours and cancers were used to support co-expression network analysis. Finally, we checked the aliases of lncRNAs and replaced them with unified GENCODE annotations for each lncRNA processed in our study. Furthermore, to compile more comprehensive publication support, we also integrated publications from several other databases, including lncRNAdb, LNCipedia, NONCODE, LncRNADisease, and Lnc2Cancer. In total, we compiled 1,038 publications related to lncRNA-cancer associations in Lnc2Catlas, among which 113, 144, and 905 supported the predictions of secondary structure disruptions, lncRNA-protein interactions, and co-expression networks, respectively, covering 562 lncRNAs.

### Implementation

Lnc2Catlas is deployed on an Apache HTTP Server 2.4.7 with a Linux (Ubuntu14.04) operating system. The database was built using a Django web framework (version 1.11.1) and the MySQL database (version 5.5.55). We deposited Lnc2Catlas on Alibaba Cloud, which provides fast memory and the latest Intel CPUs to help users achieve ultra-low latency. The web interface was based on Bootstrap 3, a framework integrating HTML, CSS, and JavaScript, which can easily and efficiently scale websites on both handheld devices and older browsers (Figure S1). Graphs and charts are generated for users to interactively visualize and access the data using echarts3 (http://echarts.baidu.com/) and cytoscape.js^44^. Five MySQL tables were created to efficiently organize and store the huge amount of data in Lnc2Catlas. The first four tables store the results obtained with RNAsnp, Global Score, WGCNA, and g:Profiler. The fifth table contains basic information on the lncRNAs, including sequences, gene expression, and publication support. LncRNA transcript IDs act as a primary key in all tables (Figure S2).

## Electronic supplementary material

Supplementary Files
