# Supplementary material for: Lnc2Catlas: an atlas of long noncoding RNAs associated with risk of cancers
Source: Sci Rep. 2018 Jan 30;8:1909. doi: 10.1038/s41598-018-20232-4 (PMC5789977; doi:10.1038/s41598-018-20232-4)
Supplement: Supplementary file 1 — Supplementary Files [file 41598_2018_20232_MOESM1_ESM.pdf]

Supplementary material for

Lnc2Catlas: an atlas of long noncoding RNAs associated with risk of cancers

Chao Ren[1]†, Gaole An [1]†, Chenghui Zhao[1], Zhangyi Ouyang[1], Xiaochen Bo[1*], Wenjie Shu[1*]

[1]Department of Biotechnology, Beijing Institute of Radiation Medicine, Beijing, China

*To whom correspondence should be addressed. Tel: +86 10 6693 2211; Fax: +86 10 6821 0077; Email: shuwj@bmi.ac.cn

Correspondence may also be addressed to Xiaochen Bo. Email: boxc@bmi.ac.cn

†These authors contributed equally to this work as joint First Authors.

Supplementary figure legends

Figure S1. Mobile device-compatible interfaces of Lnc2Catlas. (A) iPhone-compatible interface. (B) iPad-compatible interface.

Figure S2. Simplified diagram of the Lnc2Catlas relational database. The blue blocks represent MySQL tables in Lnc2Catlas. The white blocks represent the columns of a certain table. The primary key is indicated by a yellow block.

Figure S3. An example illustrating how Lnc2Catlas helps reveal the potential function of a lncRNA in thyroid cancer. (A) A SNP located in the ENST00000422494.1 transcript significantly impacts the secondary structure; (B) the ENST00000422494.1 transcript is overexpressed in thyroid cancer; and (C) ENST00000422494.1 correlates with the transcript of the thyroid-cancer-related gene MPPED2 (indicated with a red box) in thyroid cancer. (D) GO and pathway analysis of genes that are highly correlated with ENST00000422494.1 involved in the cluster derived from co-expression analysis of thyroid cancer.

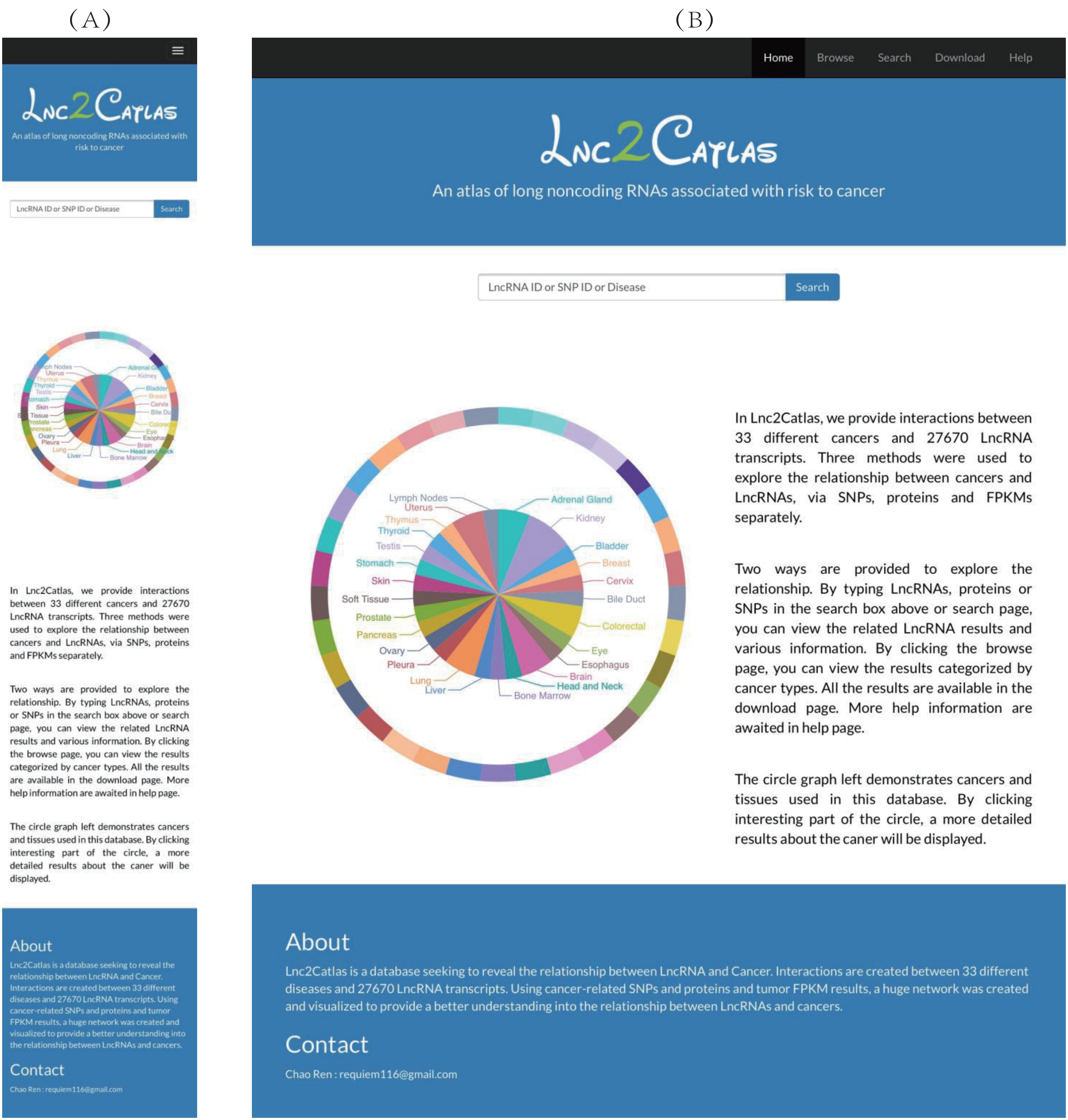
(A)
(B)
Home
Browse
Search
Download
Help
Lnc2Catlas
An atlas of long noncoding RNAs associated with risk to cancer
LncRNA ID or SNP ID or Disease
Search
Lymph Nodes
Uterus
Thymus
Thyroid
Testis
Stomach
Skin
Soft Tissue
Prostate
Pancreas
Ovary
Pleura
Lung
Liver
Adrenal Gland
Kidney
Bladder
Breast
Cervix
Bile Duct
Colorectal
Eye
Esophagus
Brain
Head and Neck
Bone Marrow
In Lnc2Catlas, we provide interactions between 33 different cancers and 27670 LncRNA transcripts. Three methods were used to explore the relationship between cancers and LncRNAs, via SNPs, proteins and FPKMs separately.
Two ways are provided to explore the relationship. By typing LncRNAs, proteins or SNPs in the search box above or search page, you can view the related LncRNA results and various information. By clicking the browse page, you can view the results categorized by cancer types. All the results are available in the download page. More help information are awaited in help page.
The circle graph left demonstrates cancers and tissues used in this database. By clicking interesting part of the circle, a more detailed results about the caner will be displayed.
About
Lnc2Catlas is a database seeking to reveal the relationship between LncRNA and Cancer. Interactions are created between 33 different diseases and 27670 LncRNA transcripts. Using cancer-related SNPs and proteins and tumor FPKM results, a huge network was created and visualized to provide a better understanding into the relationship between LncRNAs and cancers.
Contact
Chao Ren : requiem116@gmail.com

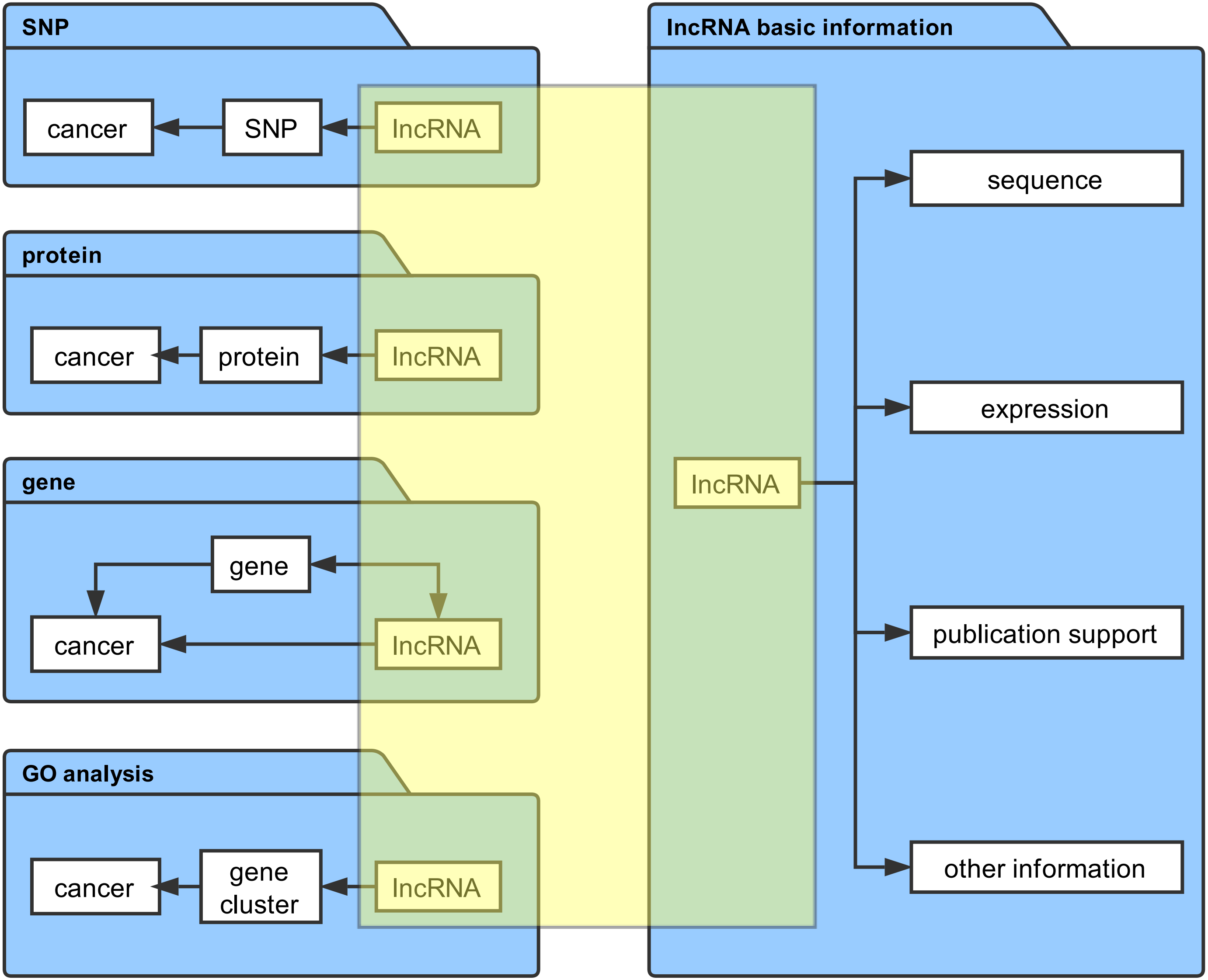
SNP
cancer
SNP
lncRNA
protein
cancer
protein
lncRNA
gene
gene
cancer
lncRNA
GO analysis
cancer
gene cluster
lncRNA
lncRNA basic information
lncRNA
sequence
expression
publication support
other information

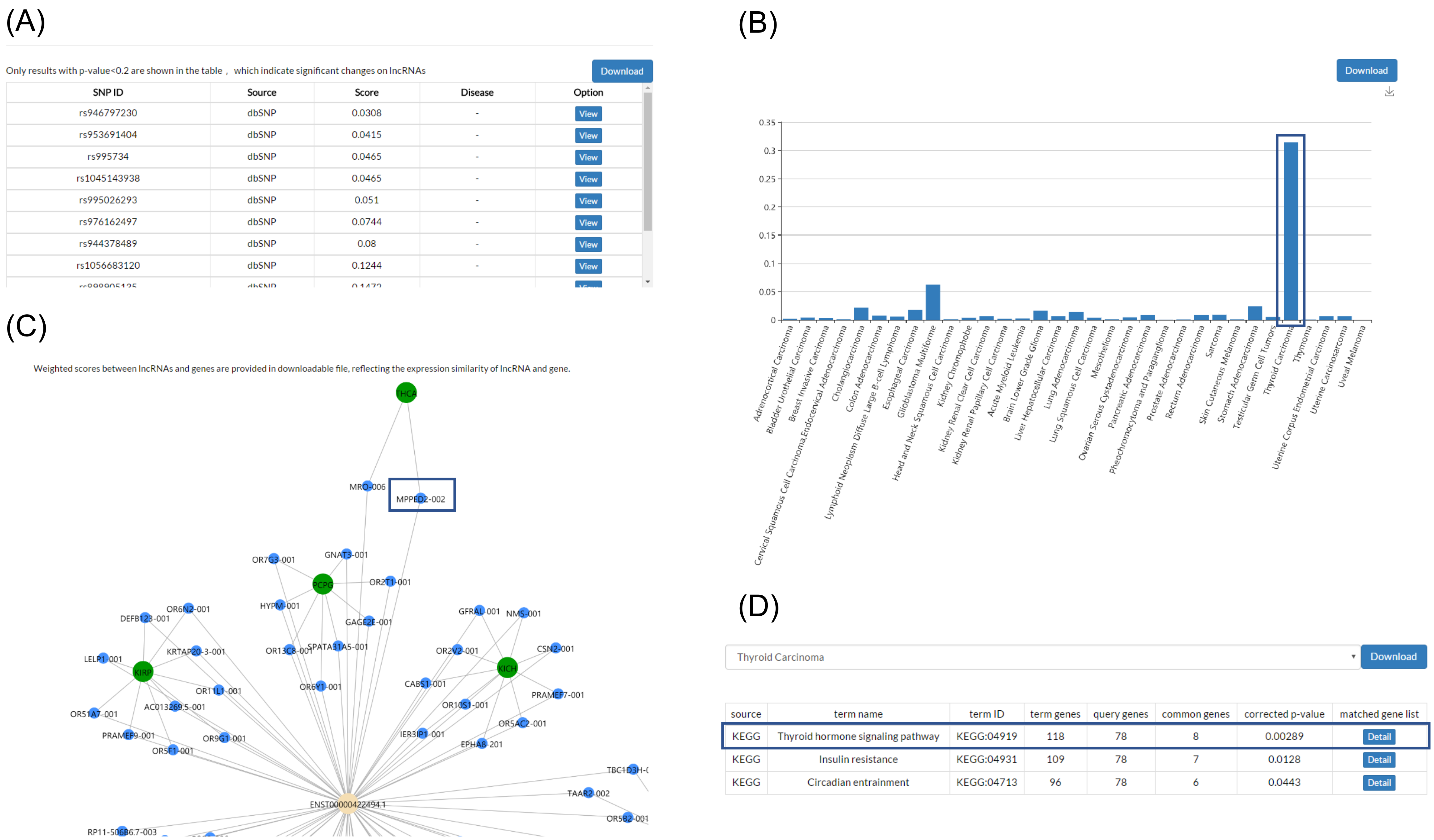
(A)
Only results with p-value<0.2 are shown in the table， which indicate significant changes on lncRNAs
Download
SNP ID
Source
Score
Disease
Option
rs946797230 dbSNP 0.0308 - View
rs953691404 dbSNP 0.0415 - View
rs995734 dbSNP 0.0465 - View
rs1045143938 dbSNP 0.0465 - View
rs995026293 dbSNP 0.051 - View
rs976162497 dbSNP 0.0744 - View
rs944378489 dbSNP 0.08 - View
rs1056683120 dbSNP 0.1244 - View
(B)
Download
0.35
0.3
0.25
0.2
0.15
0.1
0.05
0
Adrenocortical Carcinoma
Bladder Urothelial Carcinoma
Breast Invasive Carcinoma
Cervical Squamous Cell Carcinoma,Endocervical Adenocarcinoma
Cholangiocarcinoma
Colon Adenocarcinoma
Lymphoid Neoplasm Diffuse Large B-cell Lymphoma
Esophageal Carcinoma
Glioblastoma Multiforme
Head and Neck Squamous Cell Carcinoma
Kidney Chromophobe
Kidney Renal Clear Cell Carcinoma
Kidney Renal Papillary Cell Carcinoma
Acute Myeloid Leukemia
Brain Lower Grade Glioma
Liver Hepatocellular Carcinoma
Lung Adenocarcinoma
Lung Squamous Cell Carcinoma
Mesothelioma
Ovarian Serous Cystadenocarcinoma
Pancreatic Adenocarcinoma
Pheochromocytoma and Paraganglioma
Prostate Adenocarcinoma
Rectum Adenocarcinoma
Sarcoma
Skin Cutaneous Melanoma
Stomach Adenocarcinoma
Testicular Germ Cell Tumors
Thyroid Carcinoma
Thymoma
Uterine Corpus Endometrial Carcinoma
Uterine Carcinosarcoma
Uveal Melanoma
(C)
Weighted scores between lncRNAs and genes are provided in downloadable file, reflecting the expression similarity of lncRNA and gene.
THCA
MRO-006
MPPED2-002
PCPG
KIRP
KICH
ENST00000422494.1
(D)
Thyroid Carcinoma
Download
source | term name | term ID | term genes | query genes | common genes | corrected p-value | matched gene list
KEGG | Thyroid hormone signaling pathway | KEGG:04919 | 118 | 78 | 8 | 0.00289 | Detail
KEGG | Insulin resistance | KEGG:04931 | 109 | 78 | 7 | 0.0128 | Detail
KEGG | Circadian entrainment | KEGG:04713 | 96 | 78 | 6 | 0.0443 | Detail
